# COVID-19 mortality associated with multidimensional poverty, ethnicities, and armed conflict in Colombia: retrospective observational study based on national records

**DOI:** 10.3389/fpubh.2025.1514076

**Published:** 2025-05-23

**Authors:** Claudia Inés Birchenall-Jiménez, Wilson Giovanni Jiménez-Barbosa, Javier Riascos-Ochoa, Federico Cosenz

**Affiliations:** ^1^Intensive Care Department, Hospital Universitario Mayor-Mederi, Bogotá, Colombia; ^2^Escuela de Medicina y Ciencias de la Salud, Universidad del Rosario, Bogotá, Colombia; ^3^Department of Basic Sciences and Modeling, Faculty of Natural Sciences and Engineering, Universidad Jorge Tadeo Lozano, Bogotá, Colombia; ^4^Department of Political Sciences and International Relations, University of Palermo, Palermo, Italy

**Keywords:** ethnic, armed conflicts, Colombia, mortality, multidimensional poverty

## Abstract

**Background:**

The COVID-19 pandemic in Colombia exposed the interplay between multidimensional poverty, ethnic diversity, and armed conflict. This crisis worsened inequalities, disproportionately affecting Afro-Colombian and Indigenous communities already living in adverse conditions. Armed conflict further weakened social capital, limiting wellbeing in impoverished and violent regions. This study aimed to investigate the associations of poverty, ethnicity, and conflict zones with COVID-19 mortality.

**Methods:**

A retrospective observational study based on national records was conducted in Colombia from March 2020 to December 2022, in which COVID-19 cases confirmed by PCR or antigen tests were analyzed. Demographic, ethnic, and mortality data were obtained from the National Institute of Health, while poverty data were obtained from the DANE's 2018 census. Descriptive analyses, chi-square tests, and bivariate analyses were performed. A multilevel logistic regression model identified risk factors, reporting odds ratios (ORs) with 95% confidence intervals (CIs).

**Results:**

Between March 2020 and December 2022, 6,313,872 COVID-19 cases were recorded in Colombia. Afro-Colombians 2.58% and Indigenous peoples (2.75%) had higher mortality than White/Mestizo individuals did (2.24%). ZOMAC municipalities reported a mortality rate of 3.61%, and PDET municipalities reported a mortality rate of 3.20%. Multilevel analysis revealed increased mortality risks for Afro-Colombians (OR 1.14, 95% CI 1.11–1.18), Indigenous peoples (OR 1.22, 95% CI 1.17–1.28), and residents of ZOMAC (OR 1.69, 95% CI 1.66–1.72) and PDET municipalities (OR 1.34, 95% CI 1.44–1.49).

**Conclusions:**

This study highlights disparities in COVID-19 mortality influenced by ethnic, socioeconomic, and territorial factors, with a greater burden on Afro-Colombians, Indigenous peoples, and conflict zones. Public health policies must address these structural inequalities.

## 1 Introduction

The COVID-19 health emergency in Colombia has driven significant adjustments in public policies, health strategies, and community structures (1, 2). One of the major challenges facing Colombia is the need to recognize how social factors intertwine in a country characterized by a high incidence of multidimensional poverty, territorial and ethnic diversity, and prolonged armed conflict. The COVID-19 pandemic has highlighted the increased vulnerability of communities, exacerbating social inequality due to the resulting economic crisis (3, 4).

According to the literature, studies in Bogotá have linked multidimensional poverty with higher mortality rates, revealing socioeconomic, demographic, and ethnic inequities (3, 5). Additionally, a microsimulation analysis shows that the pandemic significantly increased poverty levels, affecting men and women similarly (6). On the other hand, significant reductions in employment and economic losses have been recorded in service sectors and vulnerable regions as a result of isolation measures (7), while other studies indicate that lower socioeconomic strata experience higher contagion rates and disparities in case detection (8).

During the COVID-19 pandemic, Colombia implemented a comprehensive package of economic and health measures. Economically, the government deferred taxes and relaxed fiscal regulations—granting debtors a 1-year grace period and removing negative credit reports—and introduced labor measures that allowed companies facing significant revenue drops to postpone bonus and overtime payments, complemented by subsidies covering 40% of the minimum wage. Special credit lines and grace periods for mortgages and loans were also provided, while mobility restrictions such as the suspension of domestic air travel and border closures were enforced, supported by an Emergency Mitigation Fund and international loans. Additionally, social support programs offered incentives for farmers, unemployment benefits, direct cash transfers to low-income families, and maintained educational continuity through a shift to virtual classes and student subsidies, alongside donations from senior officials. In the health sector, mandatory preventive isolation was established from March 25 until August 31, ensuring universal coverage for 97% of the population with integrated COVID-19 care, expanded testing in collaboration with university laboratories and hospitals, the setup of 5,845 ICU beds with regulated costs, and the launch of the “Coronavirus Colombia” website as a central source of information (9).

Colombia is recognized as a multicultural country with significant ethnic diversity. Multidimensional poverty and social exclusion are closely linked to ethnic identity. Afro-Colombian and Indigenous communities are disproportionately affected by adverse socioeconomic conditions, including low educational levels, health and nutritional deficiencies, and limited access to basic services. This reality underscores that poverty in these communities is not merely an issue of income but also a structural lack of opportunities (10, 11).

The dynamics of armed conflict have the potential to erode social capital, limiting a society's ability to transform available resources into valuable functioning and wellbeing. This phenomenon is evident in Colombia, where an analysis conducted in the Department of Antioquia revealed a significant correlation between poverty and armed violence. Regions with high levels of multidimensional poverty overlap with areas most severely affected by the conflict, highlighting the interdependence between socioeconomic exclusion and vulnerability to armed violence (12). Since 2016, the peace agreement with the Revolutionary Armed Forces of Colombia (FARC), one of the main guerrilla groups, has focused social attention on Zones Most Affected by Armed Conflict (ZOMAC) through the creation of special areas implementing the Development Programs with a Territorial Focus (PDET) (13–15).

In Colombia, COVID-19 mortality has disproportionately affected certain population groups, emphasizing the need to understand the underlying social factors. This retrospective observational study based on national records aims to identify the associations between Colombia's Multidimensional Poverty Index (CMPI), ethnicity, ZOMAC, and COVID-19 mortality. We hypothesize that higher levels of multidimensional poverty, ethnic minority status (Afro-Colombian and Indigenous populations), and residence in ZOMAC are associated with increased COVID-19 mortality. By identifying high-risk population groups, this study will provide a more comprehensive understanding of the factors contributing to inequality in the country, which is crucial for developing targeted public health strategies and more precise preventive measures tailored to the specific needs of each community.

## 2 Methodology

The territorial organization of Colombia is structured at three levels: 32 departments, 1,122 municipalities, and territories with special regimes. Departments serve as the primary regional units with autonomy and elected authorities; municipalities, as the basic units of local government, manage public services and community policies; and certain municipalities, such as the Capital District of Bogotá, are classified as special districts to address specific urban challenges, while indigenous areas have their own self-governance regimes. This decentralized model enhances regional autonomy and citizen participation. According to the most recent DANE projections, the total population for 2022 is estimated at ~51.5 million inhabitants (16, 17).

### 2.1 Study design

A retrospective observational study based on national records was conducted from 6 March 2020 to 31 December 2022, focusing on the Colombian population. The eligibility criteria included all individuals with a confirmed COVID-19 diagnosis during the study period. The study utilized the database from the Instituto Nacional de Salud (INS) of Colombia, which integrates reports from the Public Health Surveillance System (SIVIGILA) and is updated with corrections from the Unique Registry of Affiliates and Deceased (RUAFND), administered by the Ministry of Health and DANE. This process ensures accurate classification of COVID-19-related deaths through automated recoding (IRIS), manual expert review, verbal autopsies, and medical record analyses. The integration of SIVIGILA with RUAFND allowed for the reclassification of cases based on updated diagnostic and epidemiological information, ensuring that the final mortality figures more accurately reflect the true burden of the disease in Colombia. COVID-19 diagnosis was confirmed through polymerase chain reaction (PCR) or antigen tests. However, antigen tests are less reliable compared to PCR tests. Information on the number of patients who underwent PCR vs. antigen testing is stored in separate databases, making it impossible to determine the diagnostic method used for each individual within the dataset analyzed in this study (18). Demographic, ethnic, and mortality data were extracted from the national COVID-19 database of the National Institute of Health of Colombia (INS) between 2020 and 2022. No active follow-up was conducted; data collection was based on records in the national database (18). Information on multidimensional poverty was obtained from the 2018 population census conducted by the National Department of Statistics (DANE) (19). ZOMACs were defined according to Article 236, Section 6 of Law 1819, dated 29 December 2016, as “the zones most affected by the armed conflict. ZOMACs constitute the group of municipalities considered to be the most affected by the conflict, as defined by the Ministry of Finance, the National Planning Department, and the Agency for Territorial Renewal (ART)” (14, 15).

### 2.2 Measurement of multidimensional poverty

**Figure 1:**
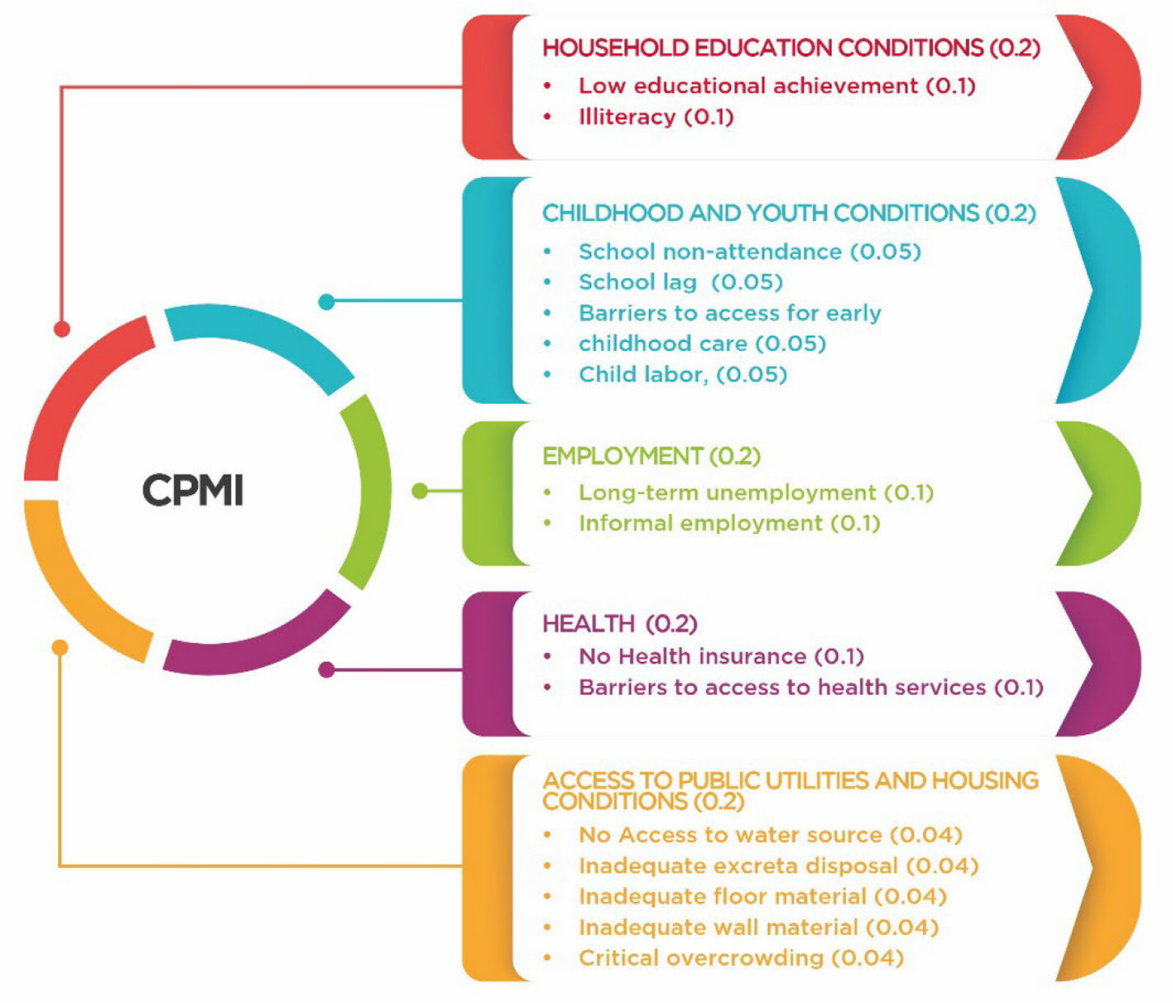
Colombian multidimensional poverty index, dimensions, and variables. Source: own elaboration.

The CMPI index was adapted by Angulo et al. on the basis of the Alkire–Foster method (20, 21). This adaptation expands the index to 5 dimensions and 15 indicators (19, 20), as shown in Figure 1 (21, 22). This index was selected because it allows for the measurement of multiple deprivations experienced by individuals simultaneously.

### 2.3 Ethnicities in Colombia

According to the 2018 National Population and Housing Census conducted by DANE, the white and mestizo population represents 88.83% of Colombia's total population, forming the largest demographic group in the country. The Afro-Colombian population, which includes Black communities, Raizales, and Palenqueros, accounts for 6.76% (2,982,224 individuals). Raizales, located in the San Andrés, Providencia, and Santa Catalina Archipelago, have Afro-Anglo-Antillean cultural roots and linguistic particularities (0.05%, 25,515 individuals), while Palenqueros, residing in San Basilio de Palenque, Bolívar, are distinguished by their Creole language and historical legacy of resistance (0.01%, 6,637 individuals). Indigenous peoples constitute 4.40% (1,905,617 individuals), maintaining their ancestral cultural and social structures across various territories in Colombia. Lastly, the Romani community, characterized by its nomadic traditions and the Romani language, represents 0.01% (2,649 individuals) (23).

### 2.4 Areas most affected by armed conflicts and municipalities with territorial focus development programs

The ZOMAC areas encompass 344 municipalities affected by armed conflict. The selection criteria included variables such as the high incidence of armed conflict and the vulnerability of these areas. Indicators of institutional weakness (lack of capacity to generate own income), multidimensional poverty (above 49%), and exclusion from major urban agglomerations in the country were used. ZOMAC areas are characterized by high levels of violence, forced displacement, and a lack of access to basic services. To address these issues, the PDET was implemented as part of the 2016 Peace Agreement with the FARC. PDET represents a comprehensive strategy to address the aftermath of armed conflict in Colombia and achieve sustainable development in the areas most affected by violence. The concept of ZOMAC originated with the 2011 Victims and Land Restitution Law, which aims to repair harm caused by the conflict (13–15, 24).

### 2.5 Data analysis

The case fatality rates (CFR) were calculated using demographic and mortality data from the National Institute of Health of Colombia (INS) COVID-19 database (18). In the present study, CFR was calculated as the proportion of deaths relative to the total number of confirmed COVID-19 cases, and then multiplied by 100 to obtain a percentage value. This indicator is essential for assessing the severity of the disease by showing the percentage of infected individuals who succumbed to the infection.

Formula:


$$\begin{array}{l} CFR=\left(\frac{Total\;deaths}{Total\;confirmed\;cases\;}\right)x\;100 \end{array}$$

Quantitative variables, including age and socioeconomic indices, were presented as medians and interquartile ranges (IQRs), ensuring comparability across all geographic areas and ethnic groups. Categorical variables, such as gender, ethnicity, PDET, and ZOMAC were presented as absolute frequencies and percentages. The normality of the quantitative variables was assessed using the Shapiro–Wilk test.

The associations between categorical variables were evaluated using the chi-square test. To compare medians between two independent groups, the nonparametric Mann–Whitney U test was used, while the Kruskal–Wallis test was employed for comparing medians between three or more independent groups. These nonparametric methods ensured comparability across diverse groups with potentially non-normally distributed data. Statistical significance was considered at α = 0.05.

A bivariate analysis was conducted to investigate associations between various independent variables and COVID-19 mortality. To analyze the association between gender and COVID-19 mortality, the Mantel-Haenszel odds ratio (OR) was calculated, stratified by age groups, to adjust for potential differences in age distribution and better isolate the effect of gender on mortality. Additionally, to evaluate the association between demographic and territorial factors and COVID-19 mortality, 2 × 2 contingency tables were constructed for the variables gender, ZOMAC classification, PDET classification, Afro-Colombian population, and Indigenous population. The OR and its corresponding 95% confidence intervals (95% CI) were estimated for each comparison, and the Pearson's chi-square test (χ^2^) was applied to assess the statistical significance of the observed associations.

For the multilevel analysis, a two-level model was employed, using municipalities as Level 1 and departments as Level 2, with mortality as the outcome variable. The database was constructed using individual variables, including age, gender, and ethnicity, while area variables were assigned to each municipality, including its corresponding CMPI as a measure of its socioeconomic conditions, as well as its classification within ZOMAC and/or PDET, as applicable. The model considered individual variables, representing personal characteristics such as age, gender, and ethnicity, and area variables, reflecting socioeconomic and territorial conditions at the municipal level. To assess variability at the departmental level while maintaining a two-level structure, the median odds ratio (MOR) was used to compare the odds ratios (OR) between municipalities and departments. The data were analyzed via STATA V.14 MP (Stata Corp.) and Excel 2019.

### 2.6 Ethical considerations

This study adhered to the ethical principles outlined in the Declaration of Helsinki-Fortaleza (Brazil 2013) and complied with Colombian regulations. Based on Resolution 8430 of 1993, Article 11, the study was classified as minimal risk. The data utilized were publicly available, anonymized, secondary sources, ensuring the protection of privacy and maintaining confidentiality.

## 3 Results

In Colombia, a total of 6,313,872 confirmed COVID-19 cases were reported from 6 March 2020 to 31 December 2022. The annual distribution of cases showed 1,746,949 cases in 2020, followed by 3,506,804 cases in 2021, and 1,060,119 cases in 2022, with a total of 142,497 deaths during the same period. The fatality rate during this period was 2.26%. Of the total confirmed COVID-19 cases, 2.94% of the mortality occurred in males, compared to 1.66% in females. Similarly, the mortality rate increased with age. Table 1 presents these findings in greater detail.

**Table 1:** COVID-19 case fatality rates by sex, age group, ethnicity, and area affected by armed conflict.

**Variable**	**Alive (%)**	**Dead (%)**	***p*-value**
**Gender**	***n* = 6,171,375**	***n* = 142,497**	
Female	98.34%	1.66%	*P* < 0.0005
Male	97.06%	2.94%	
Total	97.74%	2.26%	
Age	*n* = 6,171,375	*n* = 142,497	
< 18	99.93%	0.07%	*P* < 0.0005
18–39	99.78%	0.22%	
40–49	99.04%	0.96%	
50–59	97.62%	2.38%	
60–70	94.12%	5.88%	
70–79	87.18%	12.82%	
≥80	75.57%	24.43%	
Whites and mestizos	*n* = 5,955,808	*n* = 136,649	*P* < 0.0005
	97.76%	2.24%	
Afrocolombians	*n* = 134,079	*n* = 3,546	*P* < 0.0005
	97.42%	2.58%	
Indigenous	*n* = 80,960	*n* = 2,289	*P* < 0.0005
	97.25%	2.75%	
PDET	*n* = 373,967	*n* = 12,343	*P* < 0.0005
	96.80%	3.20%	
ZOMAC	*n* = 385,752	*n* = 14,456	*P* < 0.0005
	96.39%	3.61%	

Colombia 2020–2022.

**Figure 2:**
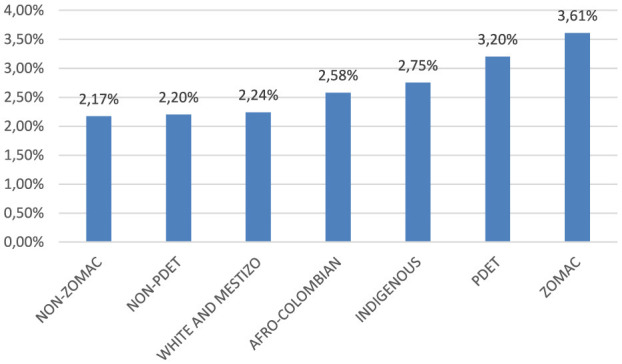
COVID-19 case fatality rate across different ethnic groups and territories affected by armed conflict. Colombia 2020–2022.

The recorded case fatality rate for the White and Mestizo populations was 2.24%. In contrast, the Afro-Colombian and Indigenous groups presented with 2.58 and 2.75%, respectively. When examining municipalities classified as ZOMAC, a rate of 3.61% was observed, whereas PDET municipalities recorded a rate 3.20%. The non-ZOMAC and non-PDET municipalities, which reported rates of 2.17 and 2.20%, respectively (Figure 2).

A comparison was made between individuals who survived and those who died from COVID-19 in Colombia, with men exhibiting a mortality rate of 2.94%, compared to 1.66% in women. Mortality increased with age, reaching 0.07% in individuals under 18 years and 24.43% in individuals aged 80 years or older. Additionally, mortality rates were 2.24% for White and Mestizo populations, 2.58% for Afro-Colombians, and 2.75% for Indigenous populations. Municipalities classified as PDET had a mortality rate of 3.20%, while those classified as ZOMAC reported a mortality rate of 3.61%. The observed differences across all categories were statistically significant with a *p*-value < 0.0005, as shown in Table 1.

The different ethnic groups showed a significant difference in the CMPI between those who survived and those who died (*p* < 0.0005), as did the PDET areas (*p* < 0.005); however, no significant difference was found in the ZOMAC areas (*p* = 0.26). The values are presented in Table 2.

**Table 2:** CMPI by race, municipality, and mortality.

**Group**	**Median CMPI (IQR)**	***P*-value**	**Median CMPI alive (IQR)**	**Median CMPI dead (IQR)**	***P*-value**
White-mestizo	12.8 (9.3)	< 0.0005	12.8 (8.6)	14.2 (14.3)	< 0.0005
Afrocolombians	19.9 (28.2)		19.9 (28.2)	21.2 (26.9)	
Indigenous	27.1 (29.6)		26.4 (29.7)	30.5 (29.1)	
Non PDET	12.8 (8.4)	< 0.0005	12.8 (5.4)	14.1 (10.3)	< 0.0005

Colombia 2020–2022.

Table 3 presents the results of the gender-adjusted bivariate analysis with the calculation of ORs. Male sex had an OR of 1.79 (CI 1.77–1.81, *p* < 0.0005), and the ORs for age increased with increasing age. An OR of 1.69 (95% CI 1.66–1.72) was observed in ZOMAC municipalities, and an OR of 1.34 (CI 1.44–1.49) was reported in PDET municipalities. The OR for Indigenous individuals was 1.22 (95% CI 1.17–1.28), and for Afro-Colombians, it was 1.14 (95% CI 1.11–1.18).

**Table 3:** Gender-adjusted bivariate analysis.

**Variable**	**OR**	**CI 95%**		***p*-value**
**Gender**				
Male (Female as the reference group**)**	1.79	1.77	1.81	< 0.0005
**Age**				
< 18	1.18	0.96	1.45	0.1064
18–39	2.12	2.01	2.23	< 0.0005
40–49	2.37	2.27	2.47	< 0.0005
50–59	2.13	2.07	2.2	< 0.0005
60–69	1.93	1.89	1.98	< 0.0005
70–79	1.87	1.83	1.91	< 0.0005
≥80	1.82	1.78	1.86	< 0.0005
ZOMAC (Non-ZOMAC as the reference group)	1.69	1.66	1.723	< 0.0005
PDET (Non-PDET as the reference group)	1.47	1.44	1.49	< 0.0005
Afrocolombians (White-Mestizo as the reference group)	1.14	1.11	1.18	< 0.0005
Indigenous (White-Mestizo as the reference group)	1.22	1.17	1.28	< 0.0005

Colombia 2020–2022.

Table 4 shows the ORs estimated from the multilevel logistic regression, which incorporates individual and area-level variables, where regional variation is observed across municipalities, with an MOR of 1.37 (95% CI 1.34–1.5, *p* < 0.0005).

**Table 4:** Multilevel analysis to determine the risk of individual- and area-level variables for COVID-19 mortality.

**Individual variable**			
**Variable**	**OR**	***p*** **-value**	**95% CI**
Age	1.09	< 0.0005	1.09–1.09
Male (Female as the reference group)	1.96	< 0.0005	1.94–1.98
Afrocolombians (White-Mestizo as the reference group)	1.10	< 0.0005	1.05–1.14
Indigenous (White-Mestizo as the reference group)	1.18	< 0.0005	1.12–1.24
Coefficient	0.0000697	< 0.0005	0.000062–0.0000783
**Area variable**			
**Variable**	**OR**	***p*** **-value**	**95% CI**
CMPI	1.003	< 0.0005	1.0015–1.0056
ZOMAC (Non-ZOMAC as the reference group)	1.11	0.008	1.02–1.20
PDET (Non-PDET as the reference group)	1.13	0.010	1.03–1.25
**Random effects and model fit**			
**Measure**	**Value**	***p*** **-value**	**95% CI**
Department variance	0.039265		0.02–0.07
ICC department	0.0114192		0.0067–0.02
MOR	1.2	< 0.0005	1.13–1.28
Municipality variance	0.109		0.095–0.126
ICC Municipality	0.043		
MOR	1.37	< 0.0005	1.34–1.40
AIC	995246.7		

Colombia 2020–2022.

## 4 Discussion

This study assesses the response to the COVID-19 pandemic in Colombia, focusing on Afro-Colombian and Indigenous communities, as well as the ZOMAC and PDET regions, which have historically been impacted by armed conflict and forced displacement. These populations, characterized by structural vulnerabilities due to limited access to healthcare services and adverse socioeconomic conditions, faced heightened precariousness during the health crisis (10, 25). The findings of this study reveal significant disparities in COVID-19 mortality in Colombia, highlighting how ethnic, socioeconomic, and territorial factors have influenced the outcomes of the pandemic. In particular, it is evident that multidimensional poverty has increased mortality rates. Afro-Colombian and Indigenous groups, along with municipalities in the ZOMAC and PDET zones, show elevated CMPI scores, which correlate with a higher risk of mortality.

In line with the literature, this study confirms that age and sex are key determinants of COVID-19 mortality. As age increases, the chance of mortality increases significantly. Similarly, the analysis revealed that men have a greater risk of mortality than women do, highlighting a notable gender disparity in pandemic outcomes (26, 27).

According to the study's results, individuals identified as Afro-Colombian and Indigenous have a 10 and 18% increased chance, respectively, compared with White-Mestizo individuals. Among the Afro-Colombian population, this finding aligns with what has been observed in countries such as Brazil and the United States, where the African American population has experienced a disproportionate impact from COVID-19, with significantly higher rates of hospitalization and mortality than other ethnic groups. This can be explained by the social vulnerability they faced prior to the pandemic; additionally, this population presents health comorbidities that contribute to the inequality they experience (28–31).

The indigenous population is also in a vulnerable position concerning COVID-19 infection. Their chance of living in poverty is three times greater than that of nonindigenous people. One of the factors contributing to increased mortality is the difficulty in adhering to social distancing measures, which heightens the chance of infection. Furthermore, preexisting health conditions, nutrition, overcrowding, and working conditions influence infection and mortality rates. Eighty-six percent of indigenous people work in the informal sector, where low wages and a lack of social security play a significant role (32). Our findings align with those reported in other studies; for example, higher COVID-19 mortality rates have been documented in this population in countries such as Brazil, whereas in Chile, their vulnerability and increased mortality have been demonstrated. Similar disparities have also been observed in the United Kingdom and the United States (33–35).

These findings must also be situated within the broader historical context of systemic inequalities rooted in Colombia's colonial legacy. Indigenous and Afro-Colombian communities have long experienced exclusion from land distribution, political participation, and equitable access to public services, while enduring persistent discrimination and racism. Their limited visibility in public discourse and transitional justice processes further reflects the deep-seated marginalization that continues to shape their realities. The COVID-19 pandemic exacerbated these structural disparities, reinforcing how historical injustices persist in influencing health outcomes and social vulnerabilities. In this context, efforts to promote health equity, memory, and social justice must incorporate decolonial and feminist perspectives that acknowledge the specific histories and lived experiences of these populations. Without adopting such critical frameworks, interventions risk perpetuating historical patterns of exclusion rather than contributing to their transformation (36).

In terms of area-level variables, even a slight rise in the CMPI is associated with a marginally higher odds of mortality, as indicated by an OR of 1.003 (95% CI: 1.0015–1.0056; *p* < 0.0005). This finding underscores the critical role of poverty as a social determinant of health. Additionally, living in the ZOMAC and PDET areas is associated with 11 and 13% increased chance, respectively, compared with living in the non-ZOMAC and non-PDET areas. This indicates that the areas most affected by conflict and territorial development initiatives continue to face significant challenges in terms of health outcomes.

The armed conflict in Colombia lasted for ~52 years, leaving behind negative consequences that impacted peace across the national territory. This prolonged situation created economic, social, and cultural challenges, significantly affecting the rural areas of the country. The border region with Venezuela continues to experience insecurity and high levels of violence perpetrated by guerrillas, paramilitary successors, and criminal groups. After the signing of the Peace Agreement in 2016, partial cessation of armed conflict was achieved, with a focus on reducing violence and managing economic and social recovery in the affected regions. This milestone marked an important step toward stabilizing and improving wellbeing in impacted areas, concentrating efforts on overcoming the challenges posed by the conflict while promoting the recovery and comprehensive development of these communities. Despite government interventions, ZOMAC and PDET municipalities face a higher chance of mortality associated with COVID-19 due to weakened healthcare systems, population displacement, and limited access to basic services. The case fatality rates highlight the vulnerability of these territories (37–39).

During the COVID-19 pandemic, the armed conflict in Colombia underwent significant transformations that reflect both a temporary de-escalation and the emergence of long-term risks. One of the main findings was the reduction in hostilities between the government and the National Liberation Army (ELN) following the unilateral ceasefire declared by the group in March 2020, as an effort to portray itself as a humanitarian actor amid the health crisis. However, the lack of a reciprocal ceasefire from the Colombian government—whose attention and military resources were redirected to pandemic response—reveals a missed opportunity to pursue sustained dialogue. At the same time, the pandemic exacerbated structural inequalities, including rising poverty and school closures, which facilitated the forced recruitment of youth by the ELN. This suggests that while armed violence temporarily subsided, deteriorating social conditions reinforced the underlying drivers of conflict. Furthermore, the ELN's strategic use of the crisis to enhance its public legitimacy—through health campaigns and aid distribution—illustrates a symbolic struggle for territorial control and political influence. Taken together, these findings indicate that the pandemic did not transform the structural roots of the conflict, but it did alter its manifestations, creating an appearance of calm that masks deep-seated tensions with the potential to resurface (40).

Innovative adaptations in health systems have become essential responses to the challenges posed by armed conflicts. Strategies such as decentralized management structures, community health worker programs, and the deployment of mobile health units have aimed to sustain service delivery under highly adverse conditions. However, persistent barriers—including infrastructure destruction, resource scarcity, and ongoing security threats—continue to severely limit effective access to essential services in affected regions. These experiences highlight not only the resilience required of health systems in crisis contexts but also the deep structural weaknesses that constrain their response capacity. Strengthening health systems in these settings remains a critical priority to achieve sustainable recovery and promote equity (41).

The variability between municipalities is significant, with an ICC suggesting that 4.3% of the total variability in the outcome is due to differences between municipalities, and an MOR of 1.37 indicates considerable disparity between municipalities. The variability at the departmental level, although smaller, remains relevant, with an ICC of 1.1% and an MOR of 1.2. These results underscore the need for public health approaches that address not only individual characteristics and ethnic disparities but also contextual factors at the municipal and departmental levels. Interventions targeting areas with high poverty rates and regions affected by conflict should be prioritized to reduce health inequalities and improve outcomes at the national level.

Beyond describing the disparities identified in COVID-19 mortality, it is important to consider the mechanisms that may underlie these outcomes. Structural inequalities—rooted in historical marginalization, systemic poverty, and restricted access to quality healthcare—likely played a central role in amplifying the pandemic's impact on Afro-Colombian, Indigenous, and conflict-affected populations. Limited access to preventive and primary care services may have delayed diagnosis and treatment, increasing the risk of severe outcomes. Furthermore, higher prevalence of chronic comorbidities such as hypertension, diabetes, and respiratory diseases—conditions often associated with social determinants of health—may have compounded vulnerability in these groups. Territorial disparities, including infrastructural deficits, scarcity of healthcare personnel, and poor health system responsiveness in ZOMAC and PDET areas, further exacerbated the risk of adverse outcomes. The interplay between socioeconomic deprivation, ethnic discrimination, and conflict-related displacements suggests that COVID-19 mortality in Colombia cannot be disentangled from long-standing social injustices. Future strategies to mitigate pandemic impacts must therefore integrate structural reforms aimed at reducing health inequities, strengthening local healthcare capacity, and addressing the broader social determinants that predispose certain populations to higher mortality.

To address the disparities in COVID-19 mortality observed among Afro-Colombian, Indigenous, and conflict-affected populations, specific policy interventions are necessary. First, strengthening primary healthcare networks in ZOMAC and PDET municipalities should be prioritized, ensuring greater access to preventive care, timely diagnosis, and management of chronic diseases. Investment in mobile health units and telemedicine could help overcome geographic barriers and improve healthcare coverage in remote and underserved areas. Second, targeted social protection programs—such as conditional cash transfers and food security initiatives—should be expanded to mitigate the socioeconomic vulnerabilities that heighten health risks during pandemics. Third, culturally sensitive public health campaigns should be developed in collaboration with Indigenous and Afro-Colombian communities to promote trust, improve health literacy, and encourage early healthcare seeking behaviors. Finally, post-conflict development programs must incorporate a health equity lens, ensuring that peace building efforts address not only economic reconstruction but also the structural health disparities that persist in conflict-affected regions. These measures are essential to reducing health inequities and building resilience against future public health emergencies.

One limitation of this study is the potential underreporting, both in the identification of ethnicities and in COVID-19 cases (32), particularly in ZOMAC areas. This situation may be due to a lack of resources, adequate healthcare infrastructure, or difficulties in data collection in regions where access is restricted due to instability. Therefore, the reported figures may not fully reflect the reality of these communities (42). Additionally, the absence of information on comorbidities in these communities adds an additional layer of complexity to the interpretation of the results.

Moreover, the study lacks information on other critical individual-level factors such as vaccination status, occupation, and access to healthcare services, all of which could influence COVID-19 outcomes. The absence of these variables introduces the possibility of residual confounding that could not be controlled for in the analysis. Another limitation lies in the reliance on secondary administrative data, which, while useful for large-scale analyses, may contain inaccuracies, missing data, or inconsistencies across different territories. In particular, ethnicity data based on self-reporting may be affected by misclassification bias, especially in contexts where ethnic identity may be underreported or inconsistently recorded.

Additionally, the use of a cross-sectional design restricts the ability to establish temporality or causal relationships between the exposure variables and COVID-19 mortality outcomes. Although associations are identified, causality cannot be definitively inferred. Furthermore, there is a risk of ecological fallacy, as area-level indicators such as CMPI or conflict exposure are attributed uniformly to individuals, even though intra-municipal variability may exist.

Finally, although multilevel models were employed to account for clustering at the municipal and departmental levels, the possibility of unmeasured spatial autocorrelation or other sources of heterogeneity remains. Future studies would benefit from longitudinal designs, more detailed clinical data, and finer geographic resolution to better elucidate the complex interplay between ethnicity, poverty, armed conflict, and health outcomes.

## 5 Conclusions

This study reveals disparities in COVID-19 mortality in Colombia, highlighting the influence of ethnic, socioeconomic, and territorial factors on pandemic outcomes. Multidimensional poverty is directly related to an increase in mortality, affecting Afro-Colombian and Indigenous groups, which were already facing adverse socioeconomic conditions before the pandemic. The historical marginalization of these communities, coupled with a lack of adequate infrastructure and resources, has exacerbated their vulnerability during the health crisis.

The significant variation in mortality rates between the ZOMAC and PDET territories compared with other regions of the country underscores the deep socioeconomic inequalities and the lasting impact of the armed conflict that persists in Colombia. These findings highlight the urgent need to design and implement public health policies that address the structural disparities that have intensified the effects of COVID-19 on the most vulnerable populations.

Importantly, these policies adopt a differential approach, recognizing and responding to the specific needs of the most affected ethnic groups and territories. Public health strategies must be inclusive and equitable, ensuring that interventions are culturally appropriate and that equitable access to healthcare resources and economic support is guaranteed. Only through an integrated and differentiated approach can the disproportionate impact of the pandemic be mitigated, and future health crises of a similar nature be prevented, thereby improving the resilience of the most vulnerable communities.

## Data Availability

The original contributions presented in the study are included in the article/supplementary material, further inquiries can be directed to the corresponding author.
